# Hospital Experience and Higher Appointments

**Published:** 1915-02-06

**Authors:** 


### HOSPITAL EXPERIENCE AND HIGHER APPOINTMENTS.

THOSE who have been following the way in which the higher administrative posts in the voluntary hospitals have been lately filled, when death or other cause has occasioned a vacancy, have noticed a tendency on the part of committees not to promote the junior hospital men, but to appoint some one who has had little or no previous hospital experience. Naturally, this has proved disappointing to the rejected hospital officers, and the question therefore arises on general grounds why the above course should have been taken. It is obvious that, other things being equal, a man who has had previous practical experience in hospital work is more likely to be fitted for the higher hospital posts than one who does not possess this qualification. It is probable, therefore, that those hospital committees which have preferred non-hospital men to junior officers when filling the higher positions, must have found disadvantages outweighing the value of previous experience in the candidates whom they passed over.

Presuming this to be the case, it becomes a legitimate, if delicate, inquiry to consider in what these disadvantages lay, and, while particular cases must be decided on the particular merits and demerits of the individual candidates, a few general considerations suggest themselves. In the first place, it is evident that for the highest lay post in a hospital certain personal qualities are needed which experience by itself cannot supply. A certain presence, a width of view, a high standard of education, and, above all, a controlling personality are necessary—all those factors, in a word, which make us say that So-and-so is a man of judgment and capacity. Now at this point it may be objected that while no training can give the magic of personality to a man not endowed therewith, yet the habit of control and authority should in its degree be inculcated among the junior men by their superiors. Yet recent events indicate that this is not necessarily the case, and any fault must be presumed to lie partly in the training given. It must be remembered that the maintenance of a high status among junior officers is always a difficult matter. For instance, the societies or organisations in which they meet, discuss, read papers, and generally occupy themselves with the theory of their profession have a difficulty incidental to their very existence. In their natural anxiety to become as representative as possible, they are apt to admit to membership, without distinction, any and every aspirant who can lay claim to official status, however humble. Now this policy, so easy to be followed, if pursued, is fatal to the society, and in the long run most detrimental to the profession as a whole. Professional organisations of this class can justify their existence only by being a training ground for their members, and that they may not degenerate into mere debating societies, or semi-social clubs, membership must be a matter of privilege. Only by this means can we be sure that all the most promising of the junior men will be attracted to it, and that consequently the training provided by discussions and meetings will maintain a high standard of interest and instruction.

It is therefore necessary that candidates for membership of such a society must not be elected as of right, merely because they have routine work to perform in some hospital department or office; they must become eligible for membership only after they have been a certain time in the profession, and achieved that sort of rank which, let us say, is comparable to that of the non-commissioned officer in the Army. To admit any and every one who can by any possibility be regarded as a hospital official is a grave mistake, and that it has been made is a deduction which the many experienced hospital men may easily draw now that junior hospital officers are not, as a matter of fact, at present gaining promotion. For it again must be repeated that the higher posts require men not merely trained in the routine of the Secretary's office, but men also of judgment, who are capable of handling affairs and also their subordinates in a statesmanlike manner worthy of the great interests which they will have to represent. All who have had experience of the onus of appointing candidates are well aware of the problem created by junior officials who imagine that a period of service alone is sufficient to raise their status, and look to time rather than capacity to secure them promotion and higher salaries. For, in the last analysis, capacity has been known to make up for want of experience; while if experience alone were enough, the policeman in the National Gallery would know more about its treasures than the Director.

